# The complete chloroplast genome and phylogeny of *Artemisia selengensis* in Dongting Lake

**DOI:** 10.1080/23802359.2018.1501322

**Published:** 2018-08-17

**Authors:** Jiao Peng, Yunlin Zhao, Chaoyang Li, Zhenggang Xu

**Affiliations:** a Hunan Research Center of Engineering Technology for Utilization of Environmental and Resources Plant, Central South University of Forestry and Technology, Changsha, People’s Republic of China;; b School of Material and Chemical Engineering, Hunan City University, Yiyang, People’s Republic of China

**Keywords:** *Artemisia selengensis*, Asteraceae, chloroplast genome, phylogeny

## Abstract

*Artemisia selengensis* Turcz (Louhao in Chinese) is a widely used health food and a well-known traditional Chinese medicine. However, only a small part of the chloroplast genome data of *Artemisia* has been reported and there was no report for *A. selengensis*. In this study, we presented the complete chloroplast genome of *A. selengensis* and analysed its phylogenetic relationship with other 28 related species belonging to the *Asteraceae* family. The result showed that the whole genome was 151 215 bp in length with a typical conserved quadripartite structure. The total GC content of whole genome, LSC, SSC, and IRa/IRb regions was 37.46, 35.55, 30.81, and 43.09%, respectively. A total of 133 genes were identified, including 88 protein-coding, 37 transfer RNA, and eight ribosome RNA genes. Among these genes, nineteen genes contained a single intron and two genes contained two introns. The phylogenetic relationship showed that *A. selengensis* was similar to *A. gmelinii*. The complete chloroplast genome presented here will enrich the genetic resources of medicinal plant and promote our understanding of the phylogeny of *Artemisia* within the Asteraceae family.

*Artemisia selengensis* Turcz (Louhao in Chinese) which belongs to the Asteraceae family is a widely used health food for its taste and nutritional properties. It has also been used as a well-known traditional Chinese medicine because some extracted substances from it have potent antitumor, antioxidant, and free radical scavenging activities (Koo et al. 1994; Shi et al. 2010). Actually, except for *A. selengensis*, many species of *Artemisia* L. are used as herbal medicines in many countries (Tariq et al. 2011). In result, the genus *Artemisia* has been the subject of genetic diversity or phylogeny studies. Previous studies have been conducted on phylogenetic relationships within *Artemisia* by using chloroplast DNA data, like *psbA*-*trnH* makers (Haghighi et al. 2014), *rps11* genes (Tariq et al. 2011), and ribosomal and chloroplast sequences (Pellicer and Garnatje 2011). However, only a fraction of the chloroplast genome data of *Artemisia* has been reported and there was no report for *A. selengensis*. Therefore, we presented the complete chloroplast genome of *A. selengensis* to enrich the genetic resources of medicinal plant and to have a comprehensive knowledge on the phylogeny of *Artemisia*.

The mature leaves of *A. selengensis* were collected from a natural population in Dongting Lake region (28°35′6.29″N and 112°20′6.29″E) and stored at −80 °C in Hunan Research Center of Engineering Technology for Utilization of Environmental and Resources Plant with accession number 20171105AS. The total chloroplast DNA was extracted by using Plant Chloroplast Purification Kit (BTN120308) and Column Plant DNA Extraction Kit and sequenced by using Illumima Hiseq 4000 Platform with Zhang’s method (Zhang et al. 2017). The raw data was filtered using Trimmomatic version 0.32 (Aachen, Germany, Bolger et al. 2014) to obtain clean data for subsequent analysis. The annotation of the whole genome and drawing of a circular map were generated by using DOGMA (Wyman et al. 2004) and OGDRAW (Lohse et al. 2007), respectively. The complete chloroplast genome was submitted to the NCBI database under the accession number MH042532.

The complete chloroplast genome of *A. selengensis* which contained a typical conserved quadripartite structure, with a LSC region of 82 920 bp, a SSC region of 18 367 bp, and a pair of IRs regions of 24 964 bp, was 151 215 bp in length. The total GC content of whole genome, LSC, SSC, and IRa/IRb regions was 37.46, 35.55, 30.81, and 43.09%, respectively. A total of 133 genes were identified, including 88 protein-coding, 37 transfer RNA, and eight ribosome RNA genes. Among these genes, nineteen genes (*rps16*, *rpoC1*, *atpF*, *petB*, *petD*, *rpl16*, *rpl2* × 2, *ndhB* × 2, *ndhA*, *ycf3*, *clpP*, *trnK-UUU*, *trnG-UCC*, *trnL-UAA*, *trnV-UAC*, *trnI-GAU* × 2, and *trnA-UGC* × 2) contained a single intron and two genes (*ycf3* and *clpP*) contained two introns. We further analysed phylogenetic relationship of *A. selengensis* with other 28 related species belonging to the Asteraceae family based on 72 shared protein-coding sequence by using neighbour-joining (NJ) method in MEGA version 7.0 (Arizona, USA, Figure 1) (Kumar et al. 2016). Our result confirmed that *A. selengensis* was similar to *A. gmelinii*. In all, the complete chloroplast genome presented here will enrich the genetic resources of medicinal plant and promote our understanding of the phylogeny of *Artemisia* within the Asteraceae family.

**Figure 1.**
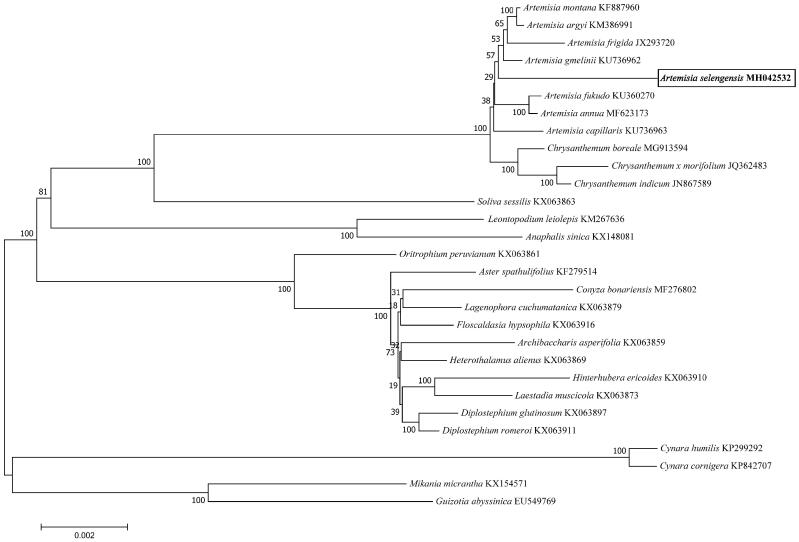
Phylogenetic tree of the relationships between *A. selengensis* and other 28 related species belonging to the Asteraceae family based on 72 shared protein-coding sequences. Branch lengths and topologies came from the neighbour-joining (NJ) analyses.
